# Promoting equity in maternal, newborn and child health – how does gender factor in? Perceptions of public servants in the Ethiopian health sector

**DOI:** 10.1080/16549716.2019.1704530

**Published:** 2020-01-14

**Authors:** Nicole Bergen, Grace Zhu, Shifera Asfaw Yedenekal, Abebe Mamo, Lakew Abebe Gebretsadik, Sudhakar Morankar, Ronald Labonté

**Affiliations:** a Faculty of Health Sciences, University of Ottawa, Ottawa, Canada; b Faculty of Medicine, University of Ottawa, Ottawa, Canada; c Department of Health, Behavior & Society, Institute of Health, Jimma University, Jimma, Ethiopia; d School of Epidemiology and Public Health, Faculty of Medicine, University of Ottawa, Ottawa, Canada

**Keywords:** Gender and Health Inequality, Determinant of health, gender barriers, gender equality, gender roles, health equity, safe motherhood, sub-Saharan Africa

## Abstract

**Background**: Advancing gender equality and health equity are concurrent priorities of the Ethiopian health sector. While gender is regarded as an important determinant of health, there is a paucity of literature that considers the interface between how these two priorities are pursued.

**Objective**: This article explores how government stakeholders understand gender issues (gender barriers and roles) in the promotion of maternal, newborn and child health equity in Ethiopia.

**Methods**: Adopting an exploratory qualitative case study design, we conducted semi-structured interviews with 17 purposively-selected stakeholders working in leadership positions with the Federal Ministry of Health and Federal Ministry of Women and Children Affairs as part of a larger study regarding the promotion of health equity in maternal, newborn and child health. A post hoc content and thematic sub-analysis was done to explore how participants raised gender issues in conversations about health equity.

**Results**: Efforts to address gender inequalities were synonymous with the promotion of a women’s health agenda, which was largely oriented towards promoting health service use. Men were predominant decision makers with regards to women’s health and health care seeking in both public and private spheres. Participants reported persisting gender-related barriers to health stemming from traditional gender roles, and noted the increased inclusion of women in the health workforce since the introduction of the Health Extension Program.

**Conclusions**: The framing of gender as a women’s health issue, advanced through patriarchal structures, does little to elevate the status of women, or promote power differentials that contribute to health inequity. Encouraging leadership roles for women as health decision makers and redressing certain gender-based norms, attitudes, practices and discrimination are possible ways forward in re-orienting gender equality efforts to align with the promotion of health equity.

## Background

**Figure 1.**
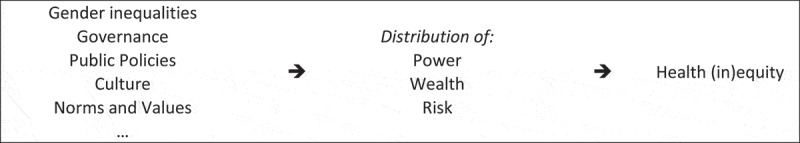
Simplified depiction of select drivers of health inequity [5,6]

Gender equality, the absence of discrimination on the basis of sex/gender [1], has steadily gained traction as a key determinant of health and a priority for global health and development. Ideally, addressing gender inequalities in health should align with and advance broader agendas to promote health equity – defined as the absence of avoidable, unfair, or remediable differences in health among subgroups of a population [2,3] – across multiple determinants of health, given that gender intersects with other determinants such as race/ethnicity, income, education, and social class [4]. In this regard, addressing the causes and consequences of gender inequalities can also shift the distribution of power, wealth, and risk within society to the benefit of disadvantaged groups (Figure 1) [5,6].

The pathways towards such a transition vary considerably (Table 1). While some approaches prioritize alleviating men-women differences in health outcomes or service use [7], others promote women’s health agendas [8], address health workforce disparities [9,10], or redefine gender norms through women’s empowerment [11]. Gender-based assessments and evaluations of programs and policies, and gender mainstreaming efforts [12] – interventions in their own right – remain integral to the advancement of gender equality. Not all approaches yield the same level of reductions in gender health inequalities, or improvements in health equity at a population scale. Assessing their effectiveness requires a grounded understanding of the dynamic conditions in which these approaches are implemented.

**Table 1.** Approaches to addressing gender inequality, with examples from Ethiopia

Approach	Brief description	Example of application in Ethiopia
Affirmative action [13]	Policies and guidelines specify opportunities for women in education or employment positions in which they are under-represented	A minimum of 5% of training and recruitment spaces are reserved for women
Gender mainstreaming [12]	Gender equality is a cross-cutting priority during the process of health development and implementation	The Federal Ministry of Health Gender Directorate seeks to facilitate gender mainstreaming at all levels of the health system [32]
Gender-based analysis [14]	Health data, monitoring, and assessments with gender equality as a primary goal	Production of sex and age disaggregated data
Gender responsive budgeting in health [32]	Allocation of resources to address gender inequality in health	Developing gender responsive budget guidelines to meet the particular health needs of men and women [15]
Health workforce initiatives [9,10]	Provide gender training and more female employment opportunities within the health sector at all levels	The Health Extension Program established an all-female cadre of community-based health workers (Health Extension Workers) to provide basic service delivery and health education [16]
Rights based [17]	Enshrine gender equality as part of human rights guaranteed by the state	Article 35 of the current 1994 Constitution of Ethiopia states that women have equal rights with men [18]
Women empowerment [11]	Support women in seeking leadership opportunities and increasing decision making power	Through the volunteer-based Women’s Development Army women are encouraged to show leadership in increasing health seeking behaviours among their neighbours
Women’s health agendas/initiatives [8]	Improve the visibility, access and quality of women-specific health services	Campaigns to increase skilled birth deliveries by providing ambulances and maternity waiting areas for rural communities

The objective of this article is to explore the intersection of gender equality and health equity in a case study of the Ethiopian health sector, specifically, the area of maternal, newborn and child health (MNCH). Ethiopia, the second-most populous country in Africa, identifies both gender equality and health equity as national policy priorities. Although separate efforts have been made to assess the state of gender equality [19,20] and monitor health equity [21,22], integrated consideration of how these two priorities are pursued is lacking. Unpacking how gender is understood as a health issue and accounted for in the promotion of health equity can begin to expose how health policies, programs, and practices can be strengthened to promote gender equality in line with Ethiopia’s broader health equity agenda.

We first identify where the country is positioned in global gender equality metrics and describe prominent efforts and commitments to address gender (i.e. gender barriers and roles) and health equity. Drawing from qualitative data gathered from public servants working at national and subnational levels, we then identify key themes related to how gender inequality is understood and confronted in the promotion of health equity within the country’s program to improve MNCH. In our analysis we highlight the complex ways that the pursuit of gender equality may both strengthen and undermine efforts to promote health equity more broadly and raise three potential health equity pitfalls of a siloed approach to conceptualizing gender.

### Gender equality and health equity in the Ethiopia context

On a global stage, gender equality was featured as one of eight Millennium Development Goals (the third goal, to promote gender equality and empower women), and was retained in the subsequent 2030 Agenda for Sustainable Development as the fifth goal (to achieve gender equality and empower all women and girls) [23,24]. Although Ethiopia tends to perform poorly on international gender equality measures, the country has reported improvements nationally [25]. Ethiopia scores low on the 2017 United Nations Development Programme Gender Inequality Index (ranking 121 out of 160 countries), though outperforms its neighboring countries largely due to relatively higher female labour force participation (77.2% of women aged 15 years and up) and share of seats in parliament held by women (37.3% in 2017, though this figure has increased since the change of government in 2018 [26]) [27]. While the federal government reports national gains in gender parity in primary school enrollment and other social and economic indicators, it emphasizes the challenges of addressing deeply rooted social norms and attitudes [19]. Women’s participation in the labour force contributes only minimally to official economic development indicators of the country, as much of their work is informal and unpaid [11]. Despite increasing representation of women in parliament, the political sphere in Ethiopia remains largely reserved for men, especially at decentralized levels [28].

Ethiopian commitment to address gender inequality in health is evidenced by a series of gender programs and policies (Table 1). The Federal Ministry of Health (FMOH) Health Sector Transformation Plan has committed to strengthen gender equality and women empowerment through a number of initiatives [29]. Notably, the country’s flagship Health Extension Program, launched in 2003, brought women into the paid health workforce and onto kebele (village) councils as community-based Health Extension Workers (HEWs) [30]. The Women’s Development Army (WDA), a group of female community members who support the work of the HEW, was established in 2010 [31]. In 2008 the Gender Directorate at the FMOH was created, and in 2013 the country released an updated health sector gender mainstreaming strategy to integrate gender into the day-to-day work of the country’s health workforce [32]. The ramifications of these varied approaches are increasingly under debate, a point we return to later in the discussion.

Ethiopia has a concurrent interest in promoting equity in health and development more broadly through global, regional, and national commitments. On the global stage, Ethiopia is a signatory of the United Nations 2030 Agenda for Sustainable Development, which includes equity as one of three fundamental principles (alongside human rights and sustainability) [24]. Regionally, Ethiopia is well-positioned to achieve the aspirations of the African Union 2063 Agenda, which stresses the ‘right to development and equity’ and the importance of ‘equitable access’ to financially sustainable health care systems [33,34]. Nationally, equity is explicitly stated as a priority in the FMOH’s Health Sector Transformation Plan [29] and through civil society initiatives to promote health equity [35,36].

MNCH has been a longstanding priority in Ethiopia, however, the advancement of equity in MNCH has only more recently gained political attention during the Millennium Development Goal implementation period, when the country reported large geographical and urban-rural differences in health services [37]. The health sector is advancing universal health coverage of MNCH services with the goal of eliminating all preventable childhood deaths by 2035. Accordingly, the National Strategy for Newborn and Child Survival in Ethiopia addresses equity through deploying specialized delivery strategies and approaches for certain regions and populations with specific needs, such as pastoralist and cross-border communities [38]. For instance, the Health System Special Support Directorate of the FMOH provides technical assistance to under-performing areas through an onsite presence and frequent supportive supervision [39]. The advancement of equity in MNCH in Ethiopia – especially addressing inequities that affect rural areas, where 80% of the population resides [40] – is a complex and ongoing challenge [37].

## Methods

### Study approach and design

This research is part of the Safe Motherhood Project, a mixed-methods intervention trial testing the rollout and scale up of interventions to reduce preventable maternal and newborn morbidity and mortality [41–43]. The Safe Motherhood Project has rolled out two interventions (an information, education and communication intervention, and maternity waiting area (MWA) upgrades) and conducted multiple rounds of data collection (prior to the design of the interventions, at baseline and at end line) [44]. MWAs, also known as maternal waiting homes, are residential homes near to the health facility where women can stay in the weeks before their delivery date, thereby overcoming geographic or transportation delays in getting to the health facility and increasing their rate of skilled birth delivery [41].

The findings of this article derive from a larger study undertaken in conjunction with the Safe Motherhood Project that explored the perceptions and experiences of Ethiopian government stakeholders regarding the promotion of health equity in MNCH. The study explored how health equity is conceptualized and operationalized, and how this, in turn, influences the promotion of equity in MNCH. In the absence of research about the conceptualization of health equity in this setting, an exploratory qualitative case study design was used, incorporating a review of policy documents as well as interviews with government and non-government stakeholders. The case study design is appropriate for research questions that explore the ‘how’ and ‘why’ of a phenomenon [45] (in this case, how health equity is understood and promoted by government stakeholders in Ethiopia).

This article is a sub-analysis of the interviews conducted with government stakeholders across administrative levels, including national, regional, zonal, woreda (district) and Primary Health Care Unit (PHCU) levels. The study was designed for the levels to be partially embedded at subnational levels, whereby it included subnational participants from a common region and zone, and from three woredas and PHCUs within that zone.

### Data collection

In-depth interviews were conducted with 17 stakeholders that held leadership positions within the FMOH (at national, regional, zonal and woreda and PHCU administrative levels), as well as within the Federal Ministry of Women and Children Affairs (at the national level). Participants were recruited purposively according to a sampling framework that specified the number of interviews to be conducted at national, regional, zonal, woreda and PHCU levels: the total number of interviews reflects individuals in positions of programmatic knowledge or authority relevant to the research questions, and working across these different administrative levels of the health system.

Of the 17 research participants, 15 were male and 2 were female; participants held titles of *Director, Coordinator* or *Expert* (Table 2). Excepting the five participants working at the national level, participants worked within the same region of Ethiopia.

**Table 2.** Study participant characteristics

Administrative level	Number included in study	Job titles
National	5	Director, Coordinator, Expert
Regional	2	Director, Coordinator
Zonal	2	Director, Coordinator
Woreda	5	Director, Coordinator
PHCU	3	Director
**Total**	**17**	

Interviews were semi-structured, covering: the social determinants of health; health equity in current scope of work; interface with health equity work at other administrative levels; and collaborations with sectors or groups. Although gender equity was not an explicit focus in these interviews, statements and perceptions about gender arose in these interviews. Thus, we returned to the interview data to conduct a post hoc sub-analysis, as detailed below.

All interviews at national, regional and zonal levels were conducted in English. At the woreda and PHCU levels, three interviews were conducted with the assistance of a research colleague who served as an interpreter for all or part of the interview. The researcher was briefed extensively about the study and provided verbatim, real-time interpretation during the interview [46]. Audio recordings of the interviews were transcribed. For the three interviews that were not conducted in English, the researcher who provided the interpretation re-listened to the audio recordings and reviewed the English version of the transcripts, making minor revisions as needed.

Ethical approval for the study was obtained from the University of Ottawa and Jimma University. All participants provided written informed consent for their participation in the study and agreed for their interview to be audio recorded. To uphold participant anonymity, pseudonyms were assigned to the participants and identifying information about their professional rank and position is not reported.

### Data analysis

A post hoc content and thematic sub-analysis was done to explore how participants raised gender issues in conversations about health equity. Noting that gender was not a specified focus of the research interviews but rather an idea that was raised by the participants, we returned to the data to capture how government stakeholders raised gender issues in conversations about health equity. After thorough familiarization with the content of the transcripts by reading and re-reading them, a two-stage coding approach was undertaken. Using Atlas.ti software, researchers first identified all passages that directly or indirectly addressed gender (gender-relevant quotations), yielding 126 quotations. A code guide was then developed inductively and gender-relevant quotations recoded accordingly. Summary outputs were generated for each code to explore convergent and divergent ideas, which were then combined into four overarching themes (see Table A1). The researchers reached consensus through discussion on the naming and content of the themes, as well as the fine tuning of the results.

The researchers who undertook the data analysis were also involved in other aspects of the Safe Motherhood Project, and thus the interpretation of the data was inevitably informed by their pre-understandings of the wider study context. In presenting the results of this analysis, the researchers endeavour to demonstrate how the current analysis was informed by and builds upon their previous and ongoing studies by referring to these other works as part of the results and discussion sections of this article.

## Results

The results are organized according to four overarching themes (see Table A1). While the first theme (recognizing traditional gender roles) addresses the question of how participants understand the relationship between gender and health, the three other themes (i.e. prioritizing a women’s health agenda, improving access to women’s health services at the community level, and working with community political and religious leaders) address the question of how participants understand gender equality issues in the context of promoting equity in MNCH.

### Recognizing traditional gender roles

Participants referenced different traditional gender roles: while men have a role in doing physically intensive tasks, women are more engaged in caregiving, household, and health promotion tasks. According to Ebise, a female participant, the burden for men and women of these traditional roles and responsibilities was mismatched with regards to the number of hours worked:

Males can work maybe 3 or 4 hours per day and sleep the whole other part, or staying and chewing his khat [a herbal stimulant]. The woman may work for the whole day, more like 8 or 10 hours. -Ebise

Women’s presumed domestic obligations affected their ability to seek health care, while men tended to be gatekeepers for health service use by women:

In most area it exists that women are most neglected and almost considered as a material in most, or some areas. They are considered as materials. They are not decision makers, even about their reproductive health issues. -Fikereye

Women’s lack of autonomous decision-making was further compounded by their lack of access to finances and other resources, authority over which was largely retained by their husbands, and which could influence health-seeking behaviour.

At the same time, participants expressed that the health sector could make changes to reduce access barriers embedded in traditional gendered role relations. One suggestion was to increase attention to men’s health concerns within the Health Extension Program, while more actively soliciting men’s involvement in MNCH issues. For example, Ayana explains his view that men should be allowed to stay at MWAs, which currently only accommodate women in their last stages of pregnancy:

[At the MWA, they] have to not restrict even the husband to stay with her. You have to allow the father, and if there are small children also, you have to let them stay there and feed them. That means we have to fill the needs of the whole family, especially the husbands and small children. Otherwise, it is difficult. -Ayana

Another participant raised the importance of including men in pregnant women educational conferences to ensure that women access the appropriate care and support during pregnancies:

The most popular meetings are the pregnant woman and midwife forum. During this pregnant woman and midwife forum, if male engagement is needed, we call for males and we make a discussion. We have a discussion – or the midwives make a discussion with the pregnant women and their partners. Actually, the first targets are the pregnant women. But we need the engagement and care for their wives and their partners. So we engage the males during the conferences. -Fikereye

### Prioritizing a women’s health agenda

Participants expressed that addressing gender in health meant prioritizing a women’s health agenda; that is, they spoke about investing in women’s health topics (namely MNCH) as a way to promote gender equality. They noted that MNCH is a priority in the Ethiopian health sector, receiving more attention than many other issues, both at the policy and the facility levels.

In most facilities, surprisingly, maternal and child health services are better off than other services because there is a lot of attention and a lot of focus from the government and different development partners. Everybody wants to improve maternal and child health. -Isaac

The major focus of the MNCH agenda is characterized by a biomedical orientation, in the sense that it aims to increase the use of health services with a particular emphasis on delivering at a health center (in contrast to the traditional practice of giving birth at home). The implications of encouraging facility-based delivery were discussed by several participants including Tariku, who explains how having a home-based delivery has come to be understood as shameful and dangerous:

The mother did not come [to deliver at the health facility] … Delivering at home is very shameful. And there is something dangerous if the mother delivered [at home] with a short period of [labour] time. -Tariku

While the intent of this health messaging is to decrease maternal mortality, participants expressed fairly widespread concern that the government-messaged imperative to increase the proportion of facility births has resulted in skewed reporting practices. This perception was affirmed by a quality assessment of the Health Management Information System (HMIS) in the three Jimma Zone districts thatcomprised our Safe Motherhood intervention study. Comparing HMIS maternal child health reporting with data from our own representative baselinesurvey revealed problems with HMIS records and poor agreement with our own survey data, indicating over-reporting of antenatal care, skilled birthattendance and postnatal care [47]. Facilities are strongly incentivized to demonstrate improvements which, as Ebise describes, leads to ‘false reporting’:

They [health officials] want that all women should deliver at the health centre … But the problem now is – and you see that everywhere, every kebele, every PHCU – the report indicates 75% coverage of facility delivery. It is important [for their performance reviews] that there is facility delivery. That means that there is false report … There are such kinds of problems that need to be stopped. -Ebise

Unreliable reporting can undermine efforts to understand which communities are underserved and hinder the ability to use evidence to address inequities in service coverage.

The government policy of promoting universal coverage for MNCH services was largely regarded by participants as a way to alleviate access barriers associated with payment for services. The ability to receive MNCH services for ‘free’ was generally regarded to be a positive development. A few participants, however, noted challenges with implementing the policy, namely community members’ lack of awareness about the policy and corrupt practices that erode its impact. Mustafa highlights the need to create awareness of the policy:

When we take the [woman to the health facility], the people assume that they [will be asked for] payments … but in this area there is no payment for any pregnant woman, there is no payment for immunization. We are advertising and advocating, through meetings, HEWs, health professionals […] so that at this time almost all [have come to] understand this … the acceptance and understanding is increasing. -Mustafa

Despite the stated importance of the MNCH agenda within the health sector, health centers are not always well equipped to provide quality MNCH services, lacking essential medical equipment or basic infrastructure such as electricity and water. Tahir gives an example of how infrastructure limitations at the health facility constrain the ability to provide MNCH services to the level required by FMOH guidelines:

Previously, the mothers stay for six hours after delivery at the health centre. This was one year ago. But since then […] the Ministry of Health and the Regional Health Bureau say that if a mother delivered at health centre, she must not be discharged until 24 hours [after birth]. This had an impact on our service. Why? Because there is not enough rooms for the mothers. If three mothers attend the delivery service at the health centre there is not enough rooms to attend the mothers for 24 hours. -Tahir

Tahir’s observation suggests that there is a lack of understanding of the resource limitations existing at the local scale by those crafting new program requirements at the national scale. It further, and somewhat provocatively, suggests that improving the state of health centers, especially in rural and remote areas, may not be a high priority for governments. To this end, Tedbabe describes how public (government) resources may be diverted towards other policy issues to the detriment of MNCH service provision:

… the commitment of government in those [health] areas is lacking and lagging behind because there are a lot of conflicting issues like weather, security issues, even food shortages are very rough. It’s [parts of Jimma Zone] a hunger and drought area in general. So, these other competing issues usually take the attention of the government away from [MNCH] issues and [from] making progress in these areas. -Tedbabe

Local communities at the kebele, PHCU, or woreda levels may be required to subsidize the shortfall in public funding. Commonly, communities fundraise to purchase or subsidize the costs of ambulances, which are important in MNCH for transporting women to the health facility. The MNCH component of the Health Extension Program also explicitly assumes that local communities will cover the costs of MWA construction and maintenance through a tithing scheme. For example, our research group has previously reported that the ‘one-birr-one-mother’ campaign, initiated by the health system, encourages community members to make monetary or in-kind donations such as cereals or coffee [41].

### Improving access to women’s health services at the community level

At the community level, women comprise nearly all of the MNCH workforce as midwives, HEWs, and WDA members. The purposive recruitment of women for these positions addresses gender imbalances in the formal workforce and encourages service use by pregnant women, who are generally hesitant to be attended by male health workers.

Several of our participants offered nuanced reflections about the role and work program of the HEWs, and the policies and conditions that guide their work. While HEWs were widely credited with improved health service access in rural Ethiopia, participants noted limitations in their training and scope of practice. For instance, HEWs are not currently permitted to practice in some areas within their scope; specifically, they are not permitted to administer clean and safe delivery despite having been trained in this area. This may be demotivating for HEWs (who, in our previous study, expressed a desire to use their training to assist with childbirth [48]) even as the training demands on HEWs are growing, as Isaac explains:

I think those packages [training components] have increased gradually from before … The Health Extension Program was so successful at the initial stages of the program, that other sectors of the government started using the Health Extension Worker for other initiatives in agriculture, and for political purposes … But still we tend not to address the root causes most of the time. -Isaac

This failure to address ‘root causes’ reflects how the HEW role has come to be seen by others working in the Ethiopian health system, and by community members themselves. Study participants noted that HEWs were most respected for providing clinical services (such as injections and medications), although a large part of their mandate and training is around health education, as corroborated by previous findings from our research [42]. Such education initiatives, however, are thought to be less valued by the community. Fikereye explains that this discrepancy can affect how HEWs approach their role:

HEWs are forced to concentrate on the clinical aspects of their work in order to get respect from the community. While she inserts the Implanon® [a long-lasting hormonal contraceptive] for example, or while she gives treatment for pneumonia the community sees that she is a clinician and they respect her. But if she only engages in part for, like providing education or something like that, the community does not respect her. Because maybe you can get this from the radio or something like that. -Fikereye

The WDA was frequently mentioned as a way in which women contribute to promoting health in their community. While some participants acknowledged that the effectiveness of the WDA was variable, especially if the members do not receive training or feel motivated, other participants pointed to ways that the WDA makes important contributions to the community. Generally, the WDA becomes the funnel through which the health care goals of the MNCH program, as communicated via HEWs, are shared and promoted within the larger kebele membership. Ayana, for instance, noted that the WDA provided useful feedback about community health issues to HEWs, and through HEWs to the larger health system. Commonly, the WDA helps to bridge the gap between health program goals and community context, for example, by raising money for upgraded or specialized health equipment. Hailu describes how a WDA initiative in remote, rural areas involved purchasing new stretchers for the dedicated purpose of carrying labouring women to the health facility, as the traditional stretchers had a primary purpose of transporting dead bodies:

Usually the mother doesn’t like to go with this kind of [traditional] stretcher because live people don’t use it – it is a stretcher for [carrying] dead people. So mothers, they don’t like to go using this stretcher. So they should be different, the stretcher that is used for the dead body and the stretcher that is used for the labouring mother. -Hailu

In this way, the WDA may be an important force in making health service use more amenable to local populations and in shifting traditional patriarchal gender norms (e.g. encouraging men to take on more household jobs while their wives are pregnant). As one means to elevate the status of the WDA, Eminish described a forthcoming program that would provide formal training for the WDA, including a certificate of competency and provisions for women who are illiterate.

This training is structured type of training, consisting of all the relevant packages. But it’s designed based on their needs. It’s a certified training, about 60-65 hours of training. After they finish the training, they can get a certificate of completion. -Eminish

Eminish notes, however, that this initiative, currently being piloted, will require the support of high-level leaders in the government to be successful. Further, the possibility of remunerating WDA members for this more formalized role seems unlikely.

### Working with community political and religious leaders

Community leaders, predominantly male, were regarded as important players in furthering women’s health agendas, often specifically addressing health-harmful traditional practices or social norms. As Girma, a male participant, explains:

We work by focusing specifically on women’s health issues … Directly, we organize different meetings to discuss and create awareness about the issues. So, our men are working in creating awareness for women’s organizations and societies. We work also indirectly on barriers against women, like harmful traditional practices … So, we work directly by selecting the topics or indirectly to help the health conditions of the women. -Girma

Participants identified politicians across various levels of government and religious leaders as key influencers in advancing women’s health, primarily through encouraging the use of MNCH services. Local politicians, such as kebele council members, were often regular points of contact for the Ministry of Health participants in this study. Tewdros acknowledged that, in his efforts to mobilize communities to improve the transportation options for pregnant women to access health facilities, he needed to start by getting buy-in from the kebele leaders, returning to the example of stretchers to transport pregnant women:

We started by discussing with the kebele leaders […] and other people from the district. And there, then we teach them [about purchasing] ambulances with the community [financial] contributions. They have said we are not going to take our mother or our wife using this stretcher … We recommend to them, if you are ready to contribute the money, it is better to discuss with the Regional Health Bureau and Federal Ministry of Health to buy ambulance. And after we discussed … finally we [were able to convince them] to buy an ambulance. -Tewdros

Participants also work with religious leaders (male) to change male community members’ attitudes and opinions about culturally sensitive MNCH issues. While not officially part of their mandate, participants recognized that aligning with religious leaders could help them to be successful in their work. Ayana, for instance, worked with religious leaders to change community acceptance for women receiving care from male health professionals:

They [religious leaders] help us to promote receiving health care from the health centre, especially when there are male attendants, since the community may not like to be attended by males. The religious leaders may tell them [the community] that it’s normal and that it’s not a problem to be attended by males. So, they can advise them like that. -Ayana

Similar to comments about working with the WDA, participants acknowledged that the strength of the relationships with political and religious leaders was variable across communities. Efforts to formally engage with these leaders have not been systematically established, though exist in some areas (including through the information, communication and education component of the Safe Motherhood Project).

## Discussion

### Complexities of addressing gender in the health sector

Our results demonstrate persisting gender-related barriers to health stemming from traditional gender roles (namely related to the physical and housekeeping/caretaking responsibilities held by men and women, respectively). While much research has characterized such roles in Ethiopia and other contexts [49], including previous analyses from the Safe Motherhood Project [50], we call attention to the less commonly noted finding from our study regarding the perceived importance of including men in MNCH spaces such as pregnant women conferences and MWAs. Such actions stand to improve men’s understanding of, commitment to, and involvement in three important elements of Ethiopia’s MNCH program: antenatal care, skilled birth delivery, and postnatal care. Engagement with male stakeholders is a key component of the Safe Motherhood Project intervention study; trial analyses are incomplete, but changes in a positive direction would indicate a shift in health equitable gender norms at the community level. While increased men’s engagement has shown to be promising in promoting shared MNCH decision-making in other African contexts [51], the current evidence base is insufficient to draw conclusions about the use of male involvement to improve MNCH and gender equality in low- and middle-income country settings [52,53].

The findings of this study begin to unravel the (sometimes unintended) gender-related implications of national-level MNCH policies and initiatives. The campaign to encourage facility-based childbirth aims to decrease maternal mortality, contributing to a reduction in a gendered health inequity. The potential for victim-blaming (shaming) women and their families who fail to give birth in a facility for any number of reasons, including geographic distance, lack of knowledge, or husband decision-making, however, can increase their ill-health via psycho-social stress. The use of positive education and engagement with mothers surrounding the place of childbirth, including managing expectations about the experience and outcome, is likely a better strategy to both empower women to make decisions about their health, and encourage increased MNCH service use [54,55].

Participant descriptions of the all-female HEWs and WDA members point to the complexities and contradictions in the demands of these roles and their implications for health equity. HEWs simultaneously face increased workload demands that could challenge their own health while remaining constrained in exercising their full range of skills. Yet, it is HEW’s clinical work that legitimizes them in the eyes of community members. The program elements that define their duties, meanwhile, may fail to move beyond medically-defined health issues to the socially embedded ‘root causes’ of ill-health, reducing the potential impact that HEWs and the Health Extension Program could have on improving health equity at a broader community scale [41].

The participants of this study expressed that, if working to their potential, the more broadly community-oriented WDA may hold great influence in changing certain patriarchal norms, hence promoting gender equality. Parallel focus group discussions with WDA members enumerated the multiple ways in which they supported the HEW and Health Extension Program goals. These included: convincing husbands to cease requiring heavy work by their later-stage pregnant wives; encouraging husbands to help transport their wives to the health facility for delivery; accompanying women to the health facility for delivery; assisting women during their stay at MWAs; promoting the importance of healthy eating during pregnancy; and discussing the benefits of antenatal and postnatal care [50]. At the same time, our focus group data suggests that there is still an element of ‘shaming’ or ‘punishment’ when women or their families fail to follow all of the MNCH program requirements [50], an approach that risks causing suffering or being demotivating [56,57].

### Health equity ramifications of addressing gender

Addressing gender is a priority of the Ethiopian health system which it implements in a number of ways, including through its high-profile MNCH agenda. While approaches to address gender inequality (Table 1) demonstrate certain complexities in their own right, as discussed above, what do they mean for the concurrent goal of improving health equity more broadly? In the following section, we consider how approaches to address gender in the health sector impact the drivers of health inequity, exploring three potential health equity pitfalls of addressing gender.

#### Pitfall #1, framing gender as a women’s health issue

While participants elaborate on ways that gender impacts health, gender framing appears binary in nature, primarily focusing on women in the context of reproductive health and childbearing. Gender equality, then, becomes an issue of their MNCH healthcare seeking behaviour. Yet, women around the globe are disproportionally affected by a host of other health-impacting issues such as informal caregiving, migrant worker conditions, noncommunicable disease risk factors, climate change, poverty, and domestic violence [58]. The extent to which an MNCH-focused health agenda ameliorates inequities in health risks experienced by women is therefore only partial.

Still, in sub-Saharan Africa where MNCH-related morbidity and mortality represent a large health burden, MNCH occupies a valid place on the health agenda. MNCH became a major priority for the Ethiopian health sector in the early 2000s with the adoption of the Millennium Development Goals and the introduction of the Health Extension Program [37]. The Millennium Development Goal period coincided with a steady decline in the annual number of maternal deaths (from 30,000 in 1993 to 11,000 in 2015) [59]; however, despite the achievements of the MNCH agenda over the past two decades, the notion noted by one of the participants in this study, that women are ‘neglected’ and ‘considered as materials’ persists. The adoption of a women’s health agenda does not in itself guarantee the elevated status of women.

The general absence of men and masculinities from discussions about gender and health (outside of their role in supporting women) is notable. Men have distinct health experiences from women (for example, related to higher participation in injurious activities), and are influenced by different health care seeking norms and ideals. Leaving men and masculinities out of conversations of gender and health gives credence to gender as a women’s health issue, and neglects a more complex consideration of the health differentials both between and among genders [8]; further, it upholds a sex/gender-based power differential that reinforces women occupying a position of vulnerability.

#### Pitfall #2, promoting gender equality through men doing right by women

The women’s health agenda in Ethiopia has, in large part, been developed and implemented by male leaders across various levels of the health sector. Men, in both public and private spheres, are predominant in decision-making roles with regards to women’s health and health care seeking. Meanwhile, community-level health workers are nearly all women. These gender roles are analogous to those at the household level, indicating a stagnation of gender-power relations. Further, they also reinforce gender-based wealth disparities, as the earnings of HEWs are not commensurate with the heavy workload and emotional burden of these positions [60], and WDA members are not compensated for their work (despite men being paid to do similar work) [61].

Given that men are established as health decision-makers, health workers and health sector campaigns target men as part of efforts to promote health service use, a reality that reinforces power inequities. The call for the growing involvement of men – husbands, community leaders, and religious leaders – in MNCH spaces begs certain questions regarding the extent to which their involvement risks reinforcing male decision-making authority and fails to further women empowerment, particularly if these actions are not accompanied by efforts to promote shared decision making. Patricia McFadden, African feminist and scholar, posits that the presence of men in women’s spaces reinforces notions of male custodianship and has ‘fundamental consequences for women’s sense of themselves and their visions of the future.’ [62]

Women’s empowerment measured through participation in decision making has been shown to have a positive effect on MNCH service use and outcomes: the percentage of women who used MNCH services decreased as the number of reasons that they felt justified wife-beating increased; and higher levels of empowerment have been correlated with lower under-five mortality [63]. Previous analyses from the Safe Motherhood Project assessed community-level perceptions of health equity, concluding that health inequity predominantly emanated from social exclusion, lack of awareness or knowledge, and financial poverty [64] – all conditions that may be exacerbated for women with low decision making authority and empowerment.

#### Pitfall #3, achieving gender equality through ‘box checking’

The adoption of gender mainstreaming by national health sectors has been questioned for its meaningfulness: while gender equality may be cited throughout national policy documents, this may be only superficial, have little bearing on how those policies are enacted or on the forces that hinder or promote their success, and become little more than a box-checking exercise [65]. In Ethiopia, the FMOH Health Sector Gender Mainstreaming Manual received little mention by the participants of this study, arguably warranting further study of its influence on how health programming is delivered [66].

Apart from the lack of progress towards meaningful change, a risk of adopting a box checking approach is the tendency to oversimplify gender equality issues and neglect the complex and intersectional nature of gender inequalities. Intersectional perspectives of gender inequality acknowledge within women and men diverse identities and dimensions of oppression such as social class, race/ethnicity, and education. Research from diverse disciplines underscore that the promotion of equity must include gender, but also be broader than only gender, considering other intersecting factors [67,68]. Proposing an agenda for gender mainstreaming and empowerment, the landmark 1995 Beijing Declaration and Platform for Action (to which Ethiopia is a signatory) emphasizes 12 critical areas of concern that extend well beyond health issues [19]. The Platform for Action upholds the principle of shared power and responsibility between women and men, and the full participation of women in public and private life including economic, social, cultural and political spheres.

### Strengths and limitations

In this article we derived our results from a sub-analysis that included participants working at different administrative levels of the health system. Gender issues emerged as a common topic of discussion, unprompted by the interviewers. This allowed us to identify themes related to gender with broad relevance across levels of the system. A strength of the study was the inclusion of individuals with senior level leadership positions, which offered perspectives from those with considerable influence in the health sector. We note that all but two of our participants were men, which attests to the gender bias in such positions. Alternate analyses might consider more detailed explorations of gender and health at a particular administrative level or incorporate perspectives from other stakeholders such as health workers or community members. A forthcoming analysis includes policy documents as a data source in a study of health equity in Ethiopia [69].

Our study of gender and health was conducted as a post hoc analysis, as gender was identified as a prominent idea that emerged in our study about perceptions and experiences in promoting health equity. While all participants spoke about their experiences with regards to gender issues, the researchers did not necessarily probe participants about these ideas. A limitation of this study is that some of the themes were described in more detail than others. A dedicated study about gender equality, including questions and probing about the topic, may elicit more detailed responses. We note, however, that several of the coauthors are closely familiar with gender topics in the study area and have experience researching MNCH and equity issues in Ethiopia. The researchers took measures to limit social desirability bias during data collection [70], and were cognizant of potential biases or inconsistencies introduced through the use of a language interpreter (research colleague) in some of the interviews [71]. To identify and explore potential points of misunderstanding, we worked closely with the researcher who provided the interpretation to clarify the intended meanings of participant responses.

## Conclusions

Through a case study of Ethiopia, this article illuminates how the health sector is working to advance gender equality. These efforts, primarily undertaken to advance health service utilization, include: promoting a women’s health agenda; reinforcing healthy behaviours through engaging male decision makers; increasing the presence of women in the health workforce; and challenging certain traditional gender roles. When considered alongside the drivers of health equity, our analysis demonstrates that addressing gender in the health sector is fraught with contradictions and complexities. Questioning the implications of actions to address gender on the distribution of power, wealth and risk reveals insights into ways in which the promotion of gender equality and health equity may sometimes be at odds. In particular, the implementation of a women’s health agenda through entrenched patriarchal structures does little to disrupt power differentials that contribute to health inequity. On a positive note, the diversity of approaches taken to elevate the status of women in Ethiopia, especially community mobilization of emerging female leaders through the WDA, serve as a source of cautious optimism for forthcoming shifting gender imbalances. Moving forward, we call for greater consideration of the consequences of efforts to promote gender equality on the broader advancement of health equity.

## Data Availability

The data that support the findings of this study are available from the corresponding author, NB, upon reasonable request.

## Paper context

The promotion of health equity involves redressing the forces in society that generate and distribute power, wealth and risk. In this paper, we demonstrate how efforts to promote gender equality in the health sector in Ethiopia have complex – and sometimes contradictory – implications on these drivers of health inequity. Further research attention is warranted to characterize how the pursuit of gender equality can better support wider efforts to promote equity in health.

## Appendix

**Table A1.** Coding guide applied in the second stage of data analysis

Code	Description	Number of quotes identified	Thematic category
MNCH	Aspects of maternal, newborn and child health	13	Prioritizing a women’s health agenda
Access equity	Factors, other than gender, that prevent women from accessing the same level of health care	38	
Health centre resources	Availability and quality of these resources in certain areas	25	
Traditional gender roles	The perception of differences between men and women in terms of ability, lifestyle, and expectations	11	Recognizing traditional gender roles
Male involvement	The role men have in MNCH (e.g. decision making)	11	
Empowerment	Women are encouraged to pursue opportunities to have a voice or improve social standing.	8	
Health care professionals	Health workers, both male and female, that influence the community and health; also includes training and quality of care	47	Improving access to women’s health services at the community level
Partners of health	Any non-governmental organizations or actors that are involved in health care	4	Working with community political and religious leaders
Leader influence	Influential leaders that can impact health perception, although their primary role is not dedicated to health	8	
